# The genetics of neuroticism and human values

**DOI:** 10.1111/gbb.12286

**Published:** 2016-04-06

**Authors:** George Zacharopoulos, Thomas M. Lancaster, Gregory R. Maio, David E. J. Linden

**Affiliations:** ^1^ School of PsychologyCardiff UniversityCardiffUK; ^2^ Neuroscience and Mental Health Research InstituteCardiff UniversityCardiffUK; ^3^ MRC Centre for Neuropsychiatric Genetics and GenomicsCardiff UniversityCardiffUK

**Keywords:** Genetics, human values, neuroticism, personality, polygenic score

## Abstract

Human values and personality have been shown to share genetic variance in twin studies. However, there is a lack of evidence about the genetic components of this association. This study examined the interplay between genes, values and personality in the case of neuroticism, because polygenic scores were available for this personality trait. First, we replicated prior evidence of a positive association between the polygenic neuroticism score (PNS) and neuroticism. Second, we found that the PNS was significantly associated with the whole human value space in a sinusoidal waveform that was consistent with Schwartz's circular model of human values. These results suggest that it is useful to consider human values in the analyses of genetic contributions to personality traits. They also pave the way for an investigation of the biological mechanisms contributing to human value orientations.

The beliefs people have about ideals that are important in life, their ‘values’, are reliably associated with certain personality traits (Parks‐Leduc *et al.* 2015; Rim 1984). Extending this connection, studies of twins have found that the shared variance between human values and personality has a significant heritable component (Schermer *et al.* 2008, 2011).

Schermer *et al.*'s (2008, 2011) analyses of shared genetic variance between traits and values utilized Schwartz's (1992) circular model of values. This model is supported by data from over 70 nations with a range of cross‐sectional, longitudinal and experimental methods (Maio 2010; Schwartz *et al.* 2012). The model posits the existence of 10 types of social values (Fig. 1a), with each expressing specific motives. These motives are organized along two dimensions. One dimension contrasts motives to promote the self (self‐enhancement) against motives that transcend personal interests (self‐transcendence), whereas the other dimension contrasts motives to follow the status quo (conservation) against motives to pursue personal intellectual and emotional interests in uncertain directions (openness). One important characteristic of this circumplex model is that it makes specific predictions about sinusoidal associations between social values and external variables. As shown in Fig. 1b, this sinusoidal waveform becomes evident if the values are ordered according to their positions along the value circle: an external variable that is most positively related to a particular value should manifest less positive and progressively more negative correlations until reaching the opposing value type. This prediction has received support in many studies finding that values at opposite ends of the circular model exhibit opposing relations to other judgements and behaviour (see Schwartz 1996) and in one study observing a sinusoidal pattern in relations between values and personality traits (Parks‐Leduc *et al.* 2015). This sinusoidal waveform supports the model's assumptions about latent motivational conflicts between values.

**Figure 1:**
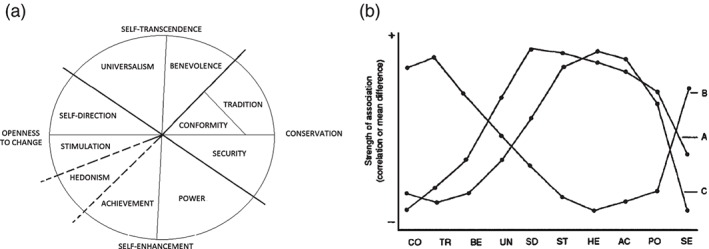
(a) The circumplex structure of personal values. (b) Plot of hypothesized relationships between three external variables (A, B and C) and the 10 values (SD, self‐direction; ST, stimulation; HE, hedonism; AC, achievement; PO, power; SE, security; CO, conformity; TR, tradition; BE, benevolence; UN, universalism). Each dot point could represent a correlation coefficient (modified from Schwartz 1992).

However, the exact genetic loci driving this association between values from Schwartz's model and personality have remained obscure. This association can be investigated by utilizing a growing body of knowledge on personality genetics. As complex psychological dispositions, human values and personality traits are both likely to be affected by numerous genes simultaneously (in addition to strong environmental influences). To capture the genetic influence of complex traits and values, it is therefore useful to focus on genetic indices that reflect the contribution of a great number of single nucleotide polymorphisms (SNPs), such as polygenic scores derived from Genome‐Wide Association Studies (GWAS).

Thus far, a polygenic score has been identified only for one trait: neuroticism. A polygenic neuroticism score (PNS) is available through a recent meta‐analysis of GWAS of personality traits (*N* = 63 661) (Genetics of Personality Consortium *et al.* 2015). Neuroticism is a personality factor ranging from emotional stability to high nervousness, tension and moodiness. In the meta‐analysis, a neuroticism score (NS) was derived from a number of measures including the NEO Personality Inventory, the Eysenck Personality Questionnaire, the International Personality Item Pool inventory, harm avoidance scores in Cloninger's Tridimensional Personality Questionnaire and negative emotionality scores in the Multidimensional Personality Questionnaire. The meta‐analysis showed that 0.6% of the variance in this NS was explained by the PNS. Although this low percentage suggests only a small genetic component, it was reliable and potentially important, making it a relevant candidate for studying genetic contributions to neuroticism and other individual differences related to neuroticism.

The shared genetic associations between personality traits and human values provide a foundation for expecting that the polygenic association with neuroticism may also relate to value orientations. Human values are particularly interesting in connection to neuroticism. A recent meta‐analysis of the relations between human values and the big five traits found reliable trait–value associations, *except* when looking at neuroticism (Parks‐Leduc *et al.* 2015). The authors explained this non‐association using Cloninger's (1994) proposition that neuroticism is more appropriately described as a temperament (i.e. an automatic associative response to emotional stimuli) than as a character trait (i.e. a self‐aware volitional concept related to behavioural intentions). This indicates a stronger biological component to neuroticism than to other traits, which, like human values, may be amenable to higher levels of cognitive processing and control. Thus, from this perspective, neuroticism may manifest a genetic component, but little association with human values.

However, a different possibility emerges if we consider relevant research examining links between neuroticism and relevant affective states and attitudes. Neuroticism is associated with a higher likelihood of anxiety and depression, which are two hallmarks of emotional instability that lead people to withdraw from the world around them (Angst *et al.* 2003; Thompson *et al.* 2011). This pattern suggests that emotional instability may cause people to be less open to new experiences, ideas and feelings, because of the potential threats to their fragile emotional state. Convergent with these observations, lower levels of neuroticism are associated with more liberal, curious and open‐minded attitudes (Carney *et al.* 2008; Van Hiel & Mervielde 2004). Strong links between such attitudes and Schwartz's openness value type (Ashton *et al.* 2005) suggest that an inverse relation between openness values (see Fig. 1a) and neuroticism is viable.

The present research was therefore motivated by the shared genetic variance between human values and personality, the existence of a polygenic score for neuroticism, and the ambiguity about neuroticism value relations. We sought to test whether the potential genetic contribution to neuroticism has similar patterns of the association with human values and the trait on a phenotypic level. To be clear, we were *not* predicting that values mediate the link between genes and traits or that traits mediate the link between genes and values. In theory, values and traits should reciprocally influence each other, as stable individual differences over time, leading to an association that is bidirectional. Our principal aim was to test whether associations with genes emerged for *both* the trait and values. Moreover, we wished to detect whether any observed associations arose in a sinusoidal pattern congruent with Schwartz's circumplex model of values.

## Materials and methods

### Subjects

A total of 81 right‐handed Caucasian university students aged between 19 and 42 (50 females; mean ± SD age = 23.85 ± 3.71) participated in the study, which was approved by the Ethics Committee in the School of Psychology, Cardiff University. Participants were informed that the study examined the connection between value–morality judgements and biological indices. They took part individually in the laboratory, wherein they completed the measures of human values and personality, provided a saliva sample, and were then debriefed. The sample used consisted of an existing sample collected for behavioural analysis. In this study we included all the participants from the existing sample for which the human value score, personality score and the genetic score were available.

### Human values

Participants completed the 56‐item Schwartz Value Survey (Schwartz 1992). Participants rated the importance of each of the 56 values as a guiding principle in their lives, using a quasi‐bipolar 9‐point scale ranging from −1 (opposed to my values), 0 (not important), 4 (important) to 7 (of supreme importance). Examples of Schwartz Value Survey items are as follows: ‘equality: equal opportunity for all’ (universalism); ‘pleasure: gratification of desires’ (hedonism); ‘obedient: dutiful meeting obligations’ (conformity). The average score across the 56 items was then calculated and subtracted from each of the 56 initial raw scores. Schwartz recommends this procedure to help control for superfluous individual variations in rating styles (Schwartz 1992). The individual centred item scores were then averaged to form scores for each type of value examined in Schwartz's model (see Fig. 1a). The internal consistency of these indices was moderate to good (see Table 1).

**Table 1:** Cronbach's α for each of the 10 values

Value	Number of items	Cronbach's *α*
Universalism	7	0.76
Benevolence	9	0.76
Tradition	6	0.63
Conformity	4	0.63
Security	6	0.68
Power	5	0.79
Achievement	6	0.67
Hedonism	2	0.74
Stimulation	3	0.79
Self‐direction	6	0.65

### Personality measure

We quantified NS using the 100‐item self‐reported version of the HEXACO Personality Inventory‐Revised (HEXACO‐PI‐R) (Lee & Ashton 2004). In the HEXACO‐PI‐R, NS is termed emotionality, and it features subscales for fearfulness, anxiety, dependence and sentimentality. These subscales are combined together as the total emotionality score (*α* = 0.64). Furthermore, many influential research programmes have interpreted and labelled neuroticism from the big five as emotional stability (De Raad *et al.* 2010; Goldberg 1990; Saucier 1994). It was previously shown that the HEXACO emotionality represents an alternative rotation of big five neuroticism (Ashton *et al.* 2014) and that they are similar constructs (Ashton *et al.* 2014; Romero *et al.* 2015). Furthermore, the emotionality score provides a particularly interesting and important rendition of neuroticism in this context because of its relative emphasis on emotional instability, which leads people to withdraw from the world around them (Angst *et al.* 2003; Thompson *et al.* 2011), and HEXACO's emotionality dimension is well suited to detecting the links with values (Pozzebon & Ashton 2009).

### DNA extraction and genotyping

Genomic DNA was obtained from saliva using Oragene OG‐500 saliva kits. Genotyping was performed using custom genotyping arrays (Illumina HumanCoreExome‐24 BeadChip), which contain 570 038 genetic variants (Illumina, Inc., San Diego, CA, USA). Quality control was implemented in plink (Purcell *et al.* 2007) to ensure that the genotypes did not display ambiguous sex, cryptic relatedness (up to third‐degree relatives by the identity of descent), genotyping completeness <97% and non‐European ethnicity admixture (detected as outliers in iterative eigenstrat analyses of an LD‐pruned data set) (Price *et al.* 2006). The SNPs were excluded where the minor allele frequency was <1%, if the call rate <98% or if the *χ* ^2^‐test for Hardy–Weinberg Equilibrium had a *P*‐value <1 e‐04. Individuals' genotypes were imputed using the pre‐phasing/imputation stepwise approach implemented in impute2/shapeit (Delaneau *et al.* 2012; Howie *et al.* 2009) and 1000Genomes (December 2013, release 1000 Genome haplotypes Phase I integrated variant set) as the reference data set.

### Generation of risk profile scores

The PNS was calculated using the method described by the International Schizophrenia Consortium (International Schizophrenia Consortium *et al.* 2009). The PNS was estimated using publicly available data from the international GWAS (Genetics of Personality Consortium *et al.* 2015). The SNPs were subsequently pruned for linkage disequilibrium (*r* ^2^ < 0.2). This method ensured that all SNPs included in the PNS model were fairly independent. The PNSs were calculated using the ‘score’ command in plink, which averages the number of risk alleles for each index SNP, weighted by the natural logarithm of the SNP's odds ratio extracted from the GWAS results (Genetics of Personality Consortium *et al.* 2015). From the 6 949 612 SNPs, a total of 206 516 quasi‐independent SNPs were considered in the PNS (PT < 0.5). We calculated PNS at the liberal *P*‐threshold (PT < 0.5), because it best predicted NS in the GWAS reference data (Genetics of Personality Consortium *et al.* 2015). There were no outliers in the PNSs, and the scores were normally distributed (Shapiro–Wilk: *P* > 0.3).

### Sinusoidal relationship analysis

To test for a sinusoidal pattern of association between values, NS and PNS, we calculated the correlation coefficients of the 10 value types with NS and PNS. The fit of the sinusoidal function presented below (eqn (1)) was calculated using the programme r.

(1) $$y=f\left(x\right)=a+b\times \sin\left(c\times x+d\right),$$

where *x* is a vector containing the correlation coefficients of the 10 values with either PNS or NS.

Firstly, all four of the parameters (*a*, *b*, *c* and *d*) of the sinusoidal function were optimized with the r command optim. The parameter *a*, the *y*‐offset, which moves the function up and down along the ordinate, was restricted to between −1 and 1, as were the correlation coefficients. The same restrictions were applied to parameter *b*, which determines the differences between the turning points of the sinusoidal function (amplitude).

The parameter *c*, the period of the sine wave, was allowed to range from 85% to 95% of a full sine wave. This restriction was based on the circular model's assumption that ‘the distances between the values around the circle may not be equal’ (Schwartz *et al.* 2012). Given that the first value type was plotted at *x* = 1, the parameter *d* (*x*‐offset), which moves the sinusoidal function along the abscissa, was set to the interval [1 + *k*/2, 1 − *k*/2]. Therefore, parameter *d* was unrestricted because there was no hypothesis regarding the exact starting point of the sine wave for each of the two measures, PNS and NS. To define a lower and upper bound given these constraints, a method developed by Byrd *et al.* (1995) was used.

We calculated the sum of the squared residuals divided by the variance to estimate the model fit indices for the sinusoidal function. This fit is called the Sinusoidal Fit Index (SFI) (Hanel *et al.* 2016) and is presented below (eqn (2)).

(2) $$SFI=\frac{\frac{1}{K-1}\;\sum_{k=1}^{K}{\left(y_{k}-\;{\hat{y}}_{k}\right)}^{2}}{\frac{1}{K-1}\sum_{k=1}^{K}{\left(y_{k}-\;{\bar{y}}_{k}\right)}^{2}}$$

In this eqn (2), *K* represents the number of correlation coefficients, *y_k_* represents the correlation coefficients, *y_k_* represents the estimated correlation coefficient through the optimization function and ${\bar{y}}_{k}$ represents the mean of the correlation coefficients. The denominator is the formula for the variance.

Hanel *et al.* (2016) tested the number of false‐positive results for the SFI, using three simulations of *m* = 100 000 samples each in r. To simulate a random pattern of correlation coefficients, they relied upon two assumptions about the distribution of the correlation coefficients. First, they sampled 10 numbers (i.e. number of human values) between −0.5 and 0.5, with *k* being the number of correlation coefficients (*k* = 10), assuming a uniform distribution. The numbers −0.5 to 0.5 represent the interval in which most correlation coefficients of values with external variables usually fall. Second, they sampled 10 numbers from a normal distribution with ∼*N*(0, 0.1) and ∼*N*(0, 0.3). Numbers >|1| were restricted to −1 or 1, respectively. For the obtained values of SFI <0.20, the percentages of false positives were below 1% for all the three simulations of 100 000 samples. The percentage of false positives for an SFI <0.20 was 0.49% (i.e. less than five false‐positive results per 1000 comparisons) assuming a normal distribution and 0.76% assuming a uniform distribution. Similarly, assuming normal and uniform distributions, respectively, the false positives were 0.20% and 0.30% for SFI <0.15. For SFI <0.10, the false positives were 0.05% and 0.08%, and for SFI <0.05, the false positives were 0.005% and 0.007%.

## Results

### Replicating the link between PNS and NS

**Figure 2:**
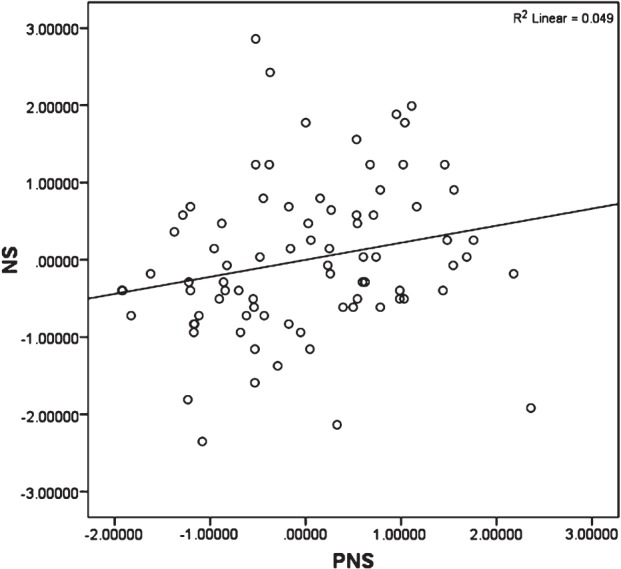
Scatter‐plot depicting the positive association between NS and PNS. Both NS (derived from the HEXACO‐PI‐R, see Material and methods) and PNS were standardized with a z‐score transformation. Each dot represents a participant.

Our first aim was to provide further evidence on the association between emotionality (NS from HEXACO‐PI‐R) and PNS. As expected, we obtained a positive association between these variables, *r*_79_ = 0.22, *P* = 0.048 (Fig. 2), replicating the findings of the personality GWAS (Genetics of Personality Consortium *et al.* 2015).

### Structure of values

Before testing for a sinusoidal waveform in the pattern of associations between values and NS, and PNS, we validated Schwartz's hypothesized circular structure in our sample. This test used two multi‐dimensional scaling (MDS) as recommended by Schwartz (Bilsky *et al.* 2011). The first analysis plotted the 56‐value items, and the second analysis plotted the 10 higher order values. Both the analyses use the respective correlation matrix to plot the values in a two‐dimensional space. The first analysis yielded S‐Stress = 0.167 and Stress I = 0.274, whereas the second analysis yielded S‐Stress = 0.032 and a Stress‐I = 0.115. The stress value is an index of how well the data fit the hypothesized configuration; higher stress values signify a poorer configuration. The stress values and the patterns in the MDS (see Table 1) were consistent with the structure hypothesized by Schwartz (1992). In addition, the openness values, self‐direction and stimulation, were significantly negatively related to NS, but these associations did not reach significance when related to the PNS.

### Fitting the sinusoidal model to the NS and PNS

**Figure 3:**
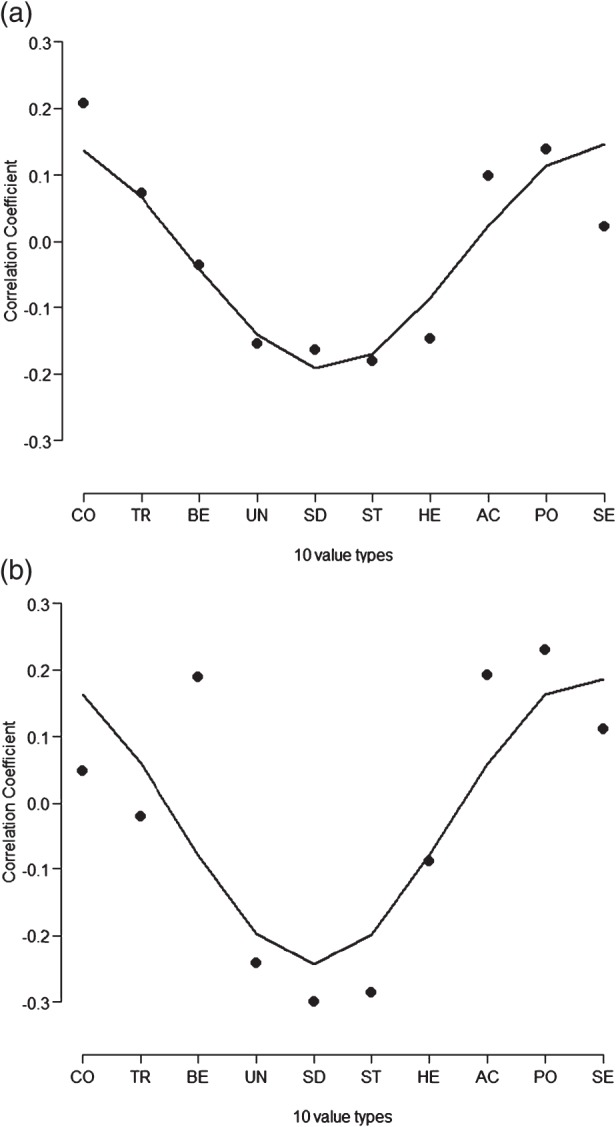
Correlation coefficients between the 10 value types (x‐axis, conformity, tradition, benevolence, universalism, self‐direction, stimulation, hedonism, achievement, power and security) and PNS (a) and NS (b).

Given our replication of Schwartz's circular structure in the MDS analyses, we turned to testing whether there are sinusoidal patterns of association between values and NS and PNS. To address this question, we plotted the correlation coefficients between NS and PNS on the *y*‐axis and each of the 10 lower order values on the *x*‐axis in an order that follows their circular structure. The patterns are shown in Fig. 3. A pattern of sinusoidal association was found between human values and PNS, particularly near the inflection points (Fig. 3a), which was significant, SFI = 0.19; false positives = 0.6%. Similarly, our analysis of NS show a sinusoidal association of a similar form (Fig. 3b) but the fit to the sine wave was not reliable, SFI = 29; *P* > 0.05. Visual inspection of Fig. 3a shows that the correlation between NS and the human value benevolence deviated from the sine wave; running the sinusoidal test while excluding benevolence yields a significant SFI = 0.08 (Fig. S1). Overall, NS and PNS map onto the human value space in similar, sinusoidal waveforms. Furthermore, in addition to testing the patterns of correlations using the SFI method, we replicated the PNS and NS findings using two previously established methods, with even more robust results (Boer & Fischer 2013; Roccas *et al.* 2002) (Appendix S1).

## Discussion

The present research investigated the genetic components connected to the relations between human values and an important dimension of personality, neuroticism. We used empirically robust measures of human values, neuroticism and genetic neuroticism. The results replicated the association between NS and PNS despite using a different measure of neuroticism than in prior research (i.e. emotionality from HEXACO‐PI‐R). This result adds to the evidence that the PNS derived by GWAS helps to explain individual variation in neuroticism (Genetics of Personality Consortium *et al.* 2015). Moreover, it laid the foundation for testing whether human values are linked to both NS and PNS. Results indicated that human values were indeed associated with NS and PNS, following the sinusoidal pattern predicted by Schwartz *et al.* (2012) cross‐cultural model.

These findings fundamentally extend the understanding of human values. Previous twin studies (Keller *et al.* 1992; Schermer *et al.* 2008, 2011; Waller *et al.* 1990) have documented that human values may have a genetic component, but this has occurred without simultaneously pinpointing relevant patterns of genes, the pattern of associations with the values and the nature of the common association to the behavioural phenotype for personality. Here, we document a novel sinusoidal relationship between human values and a specific genetic marker, the PNS – a relationship that was very similar to that found between NS and values.

Furthermore, as expected, Fig. 3 shows that the sinusoidal waveforms were anchored at one end by negative relations between values promoting stimulation or self‐direction on one hand and NS or PNS on the other hand. This pattern fits links between neuroticism and anxiety and depression. As noted earlier, anxiety and depression lead people to withdraw from the world around them (Angst *et al.* 2003; Thompson *et al.* 2011). In addition, higher levels of neuroticism are associated with less liberal, curious and open‐minded attitudes (Carney *et al.* 2008; Van Hiel & Mervielde 2004). Neuroticism may contribute to lower openness to new experiences, ideas and feelings because of the threats posed by novelty. At the same time, the pattern of withdrawal elicited by lower stimulation and self‐direction values may contribute to emotional instability by increasing rumination, perseveration in an isolated environment and self‐absorption. Further evidence is needed to explore these possibilities.

Two other aspects of our results merit further discussion. First, it is informative to contrast the sinusoidal pattern, which is a test of association across *all* values, with the strength of the correlations with *specific* values. This is interesting in part because most of the correlations between specific values and PNS or NS were weak and unreliable, aside from the significant theoretically congruent correlations discussed above (see Table S1). Nonetheless, the sinusoidal fit shows a crucial pattern that is missing from univariate tests that focus on one value at a time. It is possible for individual relations to be weak at the same time as their combined pattern is meaningful and reliable. In the analyses of values, this difference between individual correlations and the net pattern is crucial, because the relative roles of different values are psychologically more important and meaningful than the roles of any single value type in isolation, because of the competing implications between values (Rokeach 1973; Schwartz 1992).

Second, the NS variance explained from the PNS was much higher in this study (4%) than in the initial discovery sample (0.6%). A number of factors may account for the larger relation in our study. First, this study measured neuroticism using a single scale in a single homogeneous cohort, whereas the meta‐analytic study assessed neuroticism from multiple instruments (even in the same cohort). Second, this study used a single measure of neuroticism with subscales (fearfulness, anxiety, dependence and sentimentality) that are different and more emotional in focus than in the replication cohort in the meta‐analytic study (NEO‐FFI's neuroticism: anxiety, hostility, depression, self‐consciousness, impulsiveness, vulnerability to stress and Amsterdam Biographical Questionnaire). Third, the power of this study merely allows the detection of a moderate effect, and future replication studies may yield a smaller effect; therefore, future research should interpret the current effect size with caution. Despite these possibilities, the current replication of the NS–PNS relation is promising for future research attempting to learn more about this relation and its implications.

In summary, the present research (1) replicated the prior evidence of a polygenic contribution to neuroticism using a novel measure of the trait, (2) showed an association between specific genetic components and human values for the first time and (3) found a pattern of associations with values that is congruent with Schwartz's (1992) and Schwartz *et al.*'s (2012) circular model of values. Together, these results show that it is useful to include value orientations as relevant individual differences in polygenic contributions to neuroticism‐related traits, suggesting that future research should consider values in investigations of polygenic contributions to other traits.

## Supporting information


**Table S1:** Pearson correlation coefficients between PNS and NS with the 10 human values (conformity, benevolence, tradition, universalism, self‐direction, stimulation, hedonism, achievement, power and security).
**Figure S1:** Correlation coefficients between the nine value types (x‐axis, conformity, tradition, universalism, self‐direction, stimulation, hedonism, achievement, power and security) and NS.
**Appendix S1:** Replication of the sinusoidal findings of PNS and NS using two previously established methods.
