# Supplementary material for: The genetics of neuroticism and human values
Source: Genes Brain Behav. 2016 Apr 6;15(4):361–6. doi: 10.1111/gbb.12286 (PMC4950013; doi:10.1111/gbb.12286)
Supplement: Supplementary file 1 — Table S1: Pearson correlation coefficients between PNS and NS with the 10 human values (conformity, benevolence, tradition, universalism, self‐direction, stimulation, hedonism, achievement, power and security). Figure S1: Correlation coefficients between the nine value types (x‐axis, conformity, tradition, universalism, self‐direction, stimulation, hedonism, achievement, power and security) and NS. Appendix S1: Replication of the sinusoidal findings of PNS and NS using two previously established methods. [file GBB-15-361-s001.docx]

Table S1

*Cronbach’s alpha for each of the 10 values*

| Value | Number of items | Cronbach’s α |
| --- | --- | --- |
| Universalism | 7 | .76 |
| Benevolence | 9 | .76 |
| Tradition | 6 | .63 |
| Conformity | 4 | .63 |
| Security | 6 | .68 |
| Power | 5 | .79 |
| Achievement | 6 | .67 |
| Hedonism | 2 | .74 |
| Stimulation | 3 | .79 |
| Self-direction | 6 | .65 |

Table S2: Pearson Correlation coefficients between PNS and NS with the 10 human values (Conformity, Tradition, Universalism, Self-Direction, Stimulation, Hedonism, Achievement, Power, Security).

|  | **CO** | **TR** | **BE** | **UN** | **SD** | **ST** | **HE** | **AC** | **PO** | **SE** |
| --- | --- | --- | --- | --- | --- | --- | --- | --- | --- | --- |
| **PNS** | .208 | .073 | -.035 | -.155 | -.163 | -.180 | -.146 | .099 | .139 | .023 |
| **Sig. (2-tailed)** | .067 | .518 | .758 | .166 | .145 | .108 | .196 | .383 | .216 | .840 |
| **NS** | .048 | -.020 | .189 | -.242^*^ | -.299^**^ | -.285^**^ | -.088 | .192 | .230^*^ | .111 |
| **Sig. (2-tailed)** | .673 | .860 | .091 | .029 | .007 | .010 | .437 | .090 | .039 | .327 |

Figure S1

Correlation coefficients between the 9 value types (x-axis, Conformity, Tradition, Universalism, Self-Direction, Stimulation, Hedonism, Achievement, Power, Security) and NS.

Appendix S1

Apart from the sinusoidal approach discussed here, two alternative methods (Roccas et al. 2002; Boer and Fischer, 2013) for testing whether an external variable (e.g., PNS) is associated with the whole value space were utilised in the prior work. The method used in Roccas et al 2002 is composed of three steps. First, researchers derive hypotheses regarding the strongest positive and strongest negative correction between the external variable and the 10 value types. For example, in the case of extraversion discussed in their paper, it was hypothesized that the strongest positive correlation was with Stimulation and the strongest negative with Tradition. Next, researches specify the expected order of correlations by assigning numbers 1 to 10 to the ten human values with 1 assigned to the value with the strongest positive correlation and 10 to the value of the strong negative correlation following the circular model (e.g., Stimulation=1, Hedonisms and Achievement=2, Self-direction and Power=4.5, Security and Universalism=6.5, Benevolence, Conformity=8.5, Tradition=10). After this is done, the researcher performs a spearman correlation correlating the expected orders of correlations (step 2) o the observed order of correlation (i.e., empirical correlations). A significant correlation suggests that the external variable is associated with the whole value space as predicted by Schwartz’s circular model of values. By assigning the following values in step 2 (Stimulation=1, Hedonisms and Self-direction=3.5, Universalism and Achievement=6, Benevolence and Power=8, Security=9 and Conformity and Tradition=10), we replicated our PNS (r_s_=.8, p=.008) and NS (r_s_=.5, p=.112) findings using this approach. The exact same results also occur when assigning slightly different values in step 2 (Stimulation=1, Hedonisms and Self-direction=3, Universalism and Achievement=5, Benevolence and Power=7.5, Security=9 and Conformity and Tradition=10). The second approach discussed in Boer and Fischer (2013) tests 4 correlation patterns (i.e., 2 prototypical correlation patterns including the reversed patterns). The first pattern is termed SET-shape consistency and it indicates that correlations with the external variable are highest for self-transcendence values, lowest for self-enhancement values, while the correlations with other values are in between. Similarly to the first pattern, the second pattern is termed OC-shape consistency and it indicates that correlations with the external variable are highest for openness values, lowest for conservation values, while the correlations with other values are in between. Each of the correlation patterns are assigned the output statistic which is a number that ranges from -1 to 1. An absolute value greater of .4 indicates a small value consistent, .6 a medium value consistency and .8 a large value consistency. Again we replicated our finding using this approach for PNS (SET-Shape=-.40, OC-Shape=.88). However, this latter approach deemed the correlation pattern of NS also significant (SET-Shape=-.47, OC-Shape=.74).
